# A nuclear-encoded chloroplast protein harboring a single CRM domain plays an important role in the *Arabidopsis* growth and stress response

**DOI:** 10.1186/1471-2229-14-98

**Published:** 2014-04-16

**Authors:** Kwanuk Lee, Hwa Jung Lee, Dong Hyun Kim, Young Jeon, Hyun-Sook Pai, Hunseung Kang

**Affiliations:** 1 Department of Plant Biotechnology, College of Agriculture and Life Sciences, Chonnam National University, 300 Yongbong-dong, Buk-gu, Gwangju 500-757, Korea; 2 Department of Systems Biology, Yonsei University, Seoul 120-749, Korea

**Keywords:** *Arabidopsis thaliana*, Chloroplast, CRM domain, RNA-binding protein, RNA metabolism

## Abstract

**Background:**

Although several chloroplast RNA splicing and ribosome maturation (CRM) domain-containing proteins have been characterized for intron splicing and rRNA processing during chloroplast gene expression, the functional role of a majority of CRM domain proteins in plant growth and development as well as chloroplast RNA metabolism remains largely unknown. Here, we characterized the developmental and stress response roles of a nuclear-encoded chloroplast protein harboring a single CRM domain (At4g39040), designated CFM4, in *Arabidopsis thaliana*.

**Results:**

Analysis of CFM4-GFP fusion proteins revealed that CFM4 is localized to chloroplasts. The loss-of-function T-DNA insertion mutants for CFM4 (*cfm4*) displayed retarded growth and delayed senescence, suggesting that CFM4 plays a role in growth and development of plants under normal growth conditions. In addition, *cfm4* mutants showed retarded seed germination and seedling growth under stress conditions. No alteration in the splicing patterns of intron-containing chloroplast genes was observed in the mutant plants, but the processing of 16S and 4.5S rRNAs was abnormal in the mutant plants. Importantly, CFM4 was determined to possess RNA chaperone activity.

**Conclusions:**

These results suggest that the chloroplast-targeted CFM4, one of two *Arabidopsis* genes encoding a single CRM domain-containing protein, harbors RNA chaperone activity and plays a role in the *Arabidopsis* growth and stress response by affecting rRNA processing in chloroplasts.

## Background

Chloroplasts are derived from cyanobacteria through endosymbiosis, and massive gene transfer from the plastid to the nucleus occurred during evolution [1]. Chloroplasts possess approximately 100-150 genes in their own circular genome that encodes messenger RNAs, ribosome RNAs, and transfer RNAs. In addition to its own gene products, functional communication between chloroplasts and nucleus is required, and many nuclear-encoded proteins are targeted to chloroplasts and play fundamental roles in the regulation of chloroplast gene expression. Expression of chloroplast genes is commonly regulated at posttranscriptional level, including mRNA processing, splicing, editing, decay, and translational control [2-5]. A sophisticated regulatory process between chloroplasts and nuclei is required to fine-tune chloroplast gene expression, and many nuclear-encoded RNA-binding proteins (RBPs) have been recently regarded as the primary elements that modulate posttranscriptional steps in chloroplasts [2,4,6,7]. Although chloroplasts share some features of RNA metabolism with their bacterial ancestors, chloroplasts require a more complicated mechanism of RNA metabolism compared to their ancestor, which have both prokaryotic and eukaryotic characteristics [8,9]. One particular example is the splicing of group I and group II introns. In contrast to self-splicing of prokaryotic introns, chloroplasts have lost their capacity to self-splice, and the splicing of group I and group II introns in chloroplasts requires many nuclear-encoded proteins that form protein complexes similar to spliceosomal complexes found in eukaryotes [10-14]. Therefore, involvement of nuclear-encoded RBPs is indispensible for posttranscriptional regulation of RNA metabolism and gene expression in chloroplasts.

CRM (chloroplast RNA splicing and ribosome maturation) domain-containing proteins were first found in archaea and bacteria. Based on their structural data and predicted domain structures, CRM domain proteins were suggested to have RNA-binding activity [12]. Prokaryotes contain proteins harboring only a single CRM domain, whereas land plants contain proteins harboring multiple CRM domains. Splicing of group I and group II introns and tRNAs and processing of rRNAs in chloroplasts require different complexes of CRSs (for chloroplast RNA splicing), CAFs (for CRS2-associated factors), and CFMs (for CRM family members) that harbor multiple CRM domains [12,13,15-19]. It has also been demonstrated that mutation in CRM domain-containing protein genes results in pale-green phenotypes, delayed development, and aborted seed production in plants, indicating the important roles of CRM domain proteins in plant growth and development [11,12,17,20,21].

Despite an increased understanding of the roles of CRM domain-containing proteins in chloroplast RNA metabolism and plant growth and development, the functional roles of most of the CRM domain-containing proteins have not been demonstrated experimentally. The Arabidopsis (*Arabidopsis thaliana*) and rice (*Oryza sativa*) genomes harbor the genes encoding 16 and 14 CRM domain-containing proteins, respectively [13]. Among the 16 *Arabidopsis* CRM domain-containing protein genes, two genes (At4g39040 and At2g21350) encode the smallest proteins harboring a single CRM domain [13]. However, the role of single CRM domain-containing proteins has not been demonstrated in plants. Here, we determined the developmental and stress response roles of a single CRM domain-containing protein (At4g39040). Because this protein belongs to subfamily group 4 among CRM domain proteins [13], we designated it as CRM family member subfamily4 (CFM4). We show that CFM4 possesses RNA chaperone activity and is involved in rRNA processing, which is important for normal growth, development, and the stress response in plants.

## Results

### Structural features and characterization of CFM4 in *Arabidopsis*

**Figure 1:**
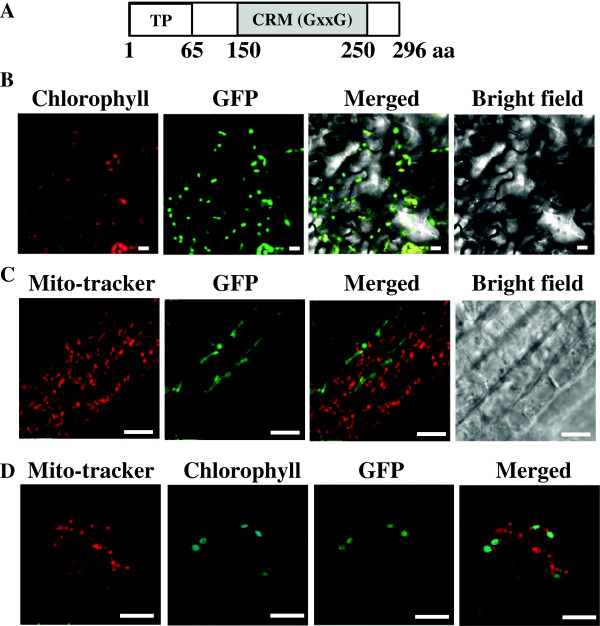
**The domain structure and cellular localization of CFM4. (A)** Schematic presentation of the domain structure of the CFM4 protein. The position of the CRM domain with a conserved GxxG sequence (gray box) is shown; TP, transit peptide. **(B)** Chloroplast localization of the CFM4 protein in *Arabidopsis* leaf. Red signals indicate chloroplast auto-fluorescence and green signals indicate GFP fluorescence. Bar = 10 μm. **(C)** Images showing mitochondria and GFP fluorescence in root. **(D)** Images showing mitochondria, chloroplast auto-fluorescence, and GFP fluorescence in leaf. Bar = 10 μm.

Sixteen predicted CRM domain family members occur in the *Arabidopsis* genome*,* and they are classified into four groups, such as CRS1 subfamily, CAF subfamily, subfamily 3, and subfamily 4. Among the 16 CRM domain-containing protein genes, two genes (At4g39040 and At2g21350) encode proteins harboring a single CRM domain and are classified into subfamily group 4 [13]. We thus named At4g39040 as CFM4. The CFM4 protein contains a highly conserved GxxG sequence in the C-terminal half of the protein (Figure 1A and Additional file 1). The two single CRM domain-containing proteins (At4g39040 and At2g21350) share approximately 56% amino acid sequence homology with each other. To examine whether the single CRM domain proteins are conserved in dicotyledonous and monocotyledonous plants, the amino acid sequences of single CRM domain proteins in diverse plant species, including *Arabidopsis*, *Zea mays*, *Medicago truncatula*, *Vitis vinifera, Hordeum vulgare, Sorghum bicolor,* and *Oryza sativa,* were compared. The results showed that CFM4 family proteins share 35-50% amino acid sequence homology among dicot and monocot plants and share > 70% amino acid sequence homology among monocot plants (Additional file 1), suggesting that the single CRM domain-containing proteins are functionally conserved in dicots and monocots.

The CRM proteins in *Arabidopsis* and rice have been predicted to be targeted mainly to chloroplasts or mitochondria. To determine the subcellular localization of CFM4, the cDNA encoding CFM4 was ligated in front of the green fluorescence protein (GFP) gene, and expression of the CFM4-GFP fusion protein was investigated in transgenic *Arabidopsis* plants. Strong GFP signals were observed in chloroplasts (Figure 1B). To examine whether CFM4 is also localized to mitochondria, *Arabidopsis* mitochondria were stained with Mito-tracker that is a red-fluorescent dye and stains mitochondria in live cells, and the signals from plastids in roots and chloroplasts in leaves were examined. The results showed that the signals from mitochondria did not overlap with the signals from chloroplasts, and GFP signals were observed exclusively in chloroplasts (Figure 1C and 1D). These results clearly indicate that CFM4 is localized to chloroplasts.

### CFM4 plays a role in *Arabidopsis* growth and senescence

**Figure 2:**
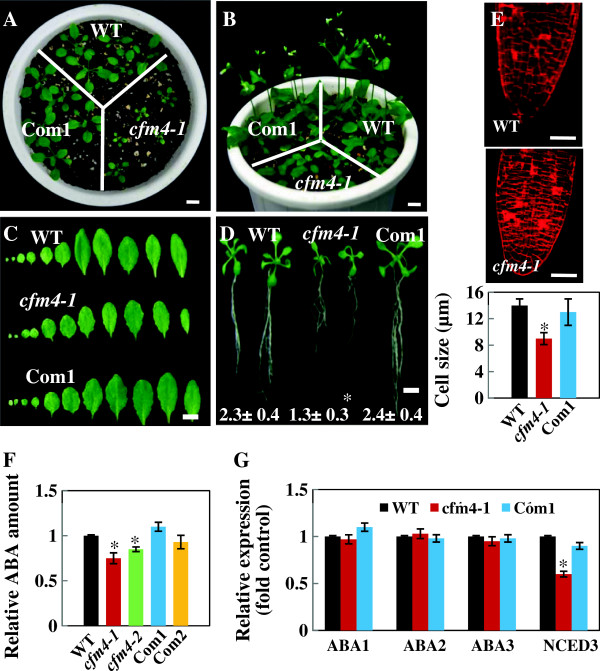
**Phenotypes of *cfm4 *mutant plants and complementation lines. (A, B)** Growth of the wild-type plants (WT), *cfm4* mutants, and complementation line (Com1) at 20 and 33 days after germination (DAG). Scale bar = 1 cm. **(C)** The size and number of leaves at emergence of floral buds on 26 DAG. Scale bar = 1 cm. **(D)** Root growth (cm) of the plants measured on 20 DAG. Scale bar = 1 cm. **(E)** The size of cells in the roots of the plants measured on 26 DAG. Scale bar = 50 μm. **(F)** The amount of ABA was measured in 26-day-old plants, and relative amount to wild-type value is shown. **(G)** Transcript levels of ABA biosynthesis-related genes, *ABA1*, *ABA2*, *ABA3*, and *NCED3*, were measured in 26-day-old plants by real-time RT-PCR, and relative expression levels to wild-type value are shown. The values are mean ± SE obtained from five independent experiments, and asterisk above the number indicates statistically different values between WT and mutant plants (*P* ≤ 0.05).

To determine the role of CFM4 during plant growth and development, the T-DNA insertion mutant lines in CFM4 (SALK_076439 and SALK_126978) were obtained, and their phenotypes were analyzed under normal and stress conditions. The absence of *CFM4* expression in the knockout mutant lines was confirmed by RT-PCR analysis (Additional file 2). The wild-type and *cfm4* mutant plants were grown in MS medium or soil, and their phenotypes were observed during the entire life cycle (from germination to senescence) of the plants. Growth of the wild-type and mutant plants was not significantly different at 7 days after germination (DAG) (Additional file 3). However, growth of the plants was markedly different at later stages in that the size of the *cfm4* mutants was much smaller than that of the wild-type plants at 20 or 23 DAG (Figure 2A and Additional file 3). The difference in flowering time between the wild-type and mutant plants was evident; *cfm4* mutants flowered approximately 7 days later than the wild-type plants (Figure 2B and Additional file 4). Although the *cfm4* mutants flowered much later than the wild-type plants, the size and number of leaves at the time of bolting were not different between the wild-type and mutant plants (Figure 2C), suggesting that CFM4 does not affect control of flowering time. No significant difference in plant height was observed between the wild-type and mutant plants at the time of maturity. The root growth of the *cfm4* mutants was also retarded compared with that of the wild-type plants (Figure 2D and Additional file 5). To determine whether the retarded root growth was due to the decreases in cell size or cell number, the plasma membranes in the roots of the wild-type and *cfm4* mutant plants were stained with FM 4-64 dye and observed under confocal microscope. The results showed that the size of cells in *cfm4* mutants was much smaller than that in the wild-type plants (Figure 2E). The retarded growth phenotypes of the mutant plants recovered to normal phenotypes in the complementation lines (Figure 2 and Additional files 4 and 5). All of these observations clearly demonstrate that CFM4 plays a role for normal growth of *Arabidopsis* plants. Because a recent study demonstrated that the growth retardation phenotypes of an *Arabidopsis* mutant are closely related to abscisic acid (ABA) biosynthesis and chloroplast RNA metabolism [22], we wanted to determine ABA levels in the wild-type plants*, cfm4* mutants, and complementation lines using an immunoassay method. The results showed that levels of ABA in *cfm4* mutants were approximately 70-80% of those in the wild-type plants (Figure 2F). We also analyzed transcript levels of the genes, including *ABA1*, *ABA2*, *ABA3*, and *NCED3* that are involved in ABA biosynthesis, and found that the levels of *NCED3* were significantly lower in *cfm4* mutants than in the wild-type plants (Figure 2G). These results suggest that the retarded growth phenotypes of *cfm4* mutants are related to impaired ABA biosynthesis.

**Figure 3:**
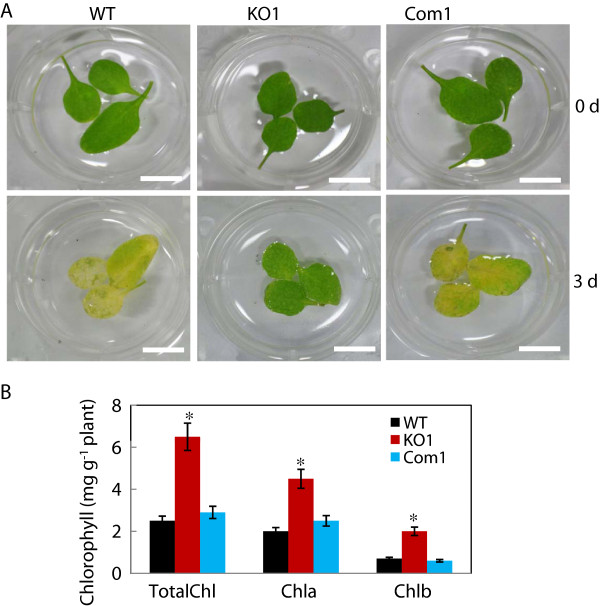
**Delayed senescence of *cfm4 *mutant plants. (A)** Rosette leaves from 4-week-old wild type (WT), *cfm4* mutants, and complementation line (Com1) were floated in water in the dark for 3 days. **(B)** Chlorophyll content was measured in the leaves of each plant 3 days after dark-induced senescence. The values are mean ± SE obtained from three independent experiments, and asterisks above the columns indicate statistically different values between WT and mutant plants (*P* ≤ 0.05).

With the observation that CFM4 plays a role in the growth of *Arabidopsis*, we subsequently examined whether CFM4 is involved in senescence. In the dark-induced senescence assay, it was evident that greening of the leaves of *cfm4* mutant plants was maintained for much longer compared with that of the wild-type plants when they were incubated under dark conditions (Figure 3A). Total chlorophyll (chlorophyll a + b) contents in *cfm4* mutant plants were much higher than those in the wild-type plants (Figure 3B). Greening of the leaves of the complementation lines was similar to that of the wild-type plants, and the chlorophyll contents in the complementation lines were comparable with those in the wild-type plants (Figure 3). These results suggest that CFM4 plays a positive role in senescence.

### CFM4 is involved in *Arabidopsis* response to abiotic stresses

**Figure 4:**
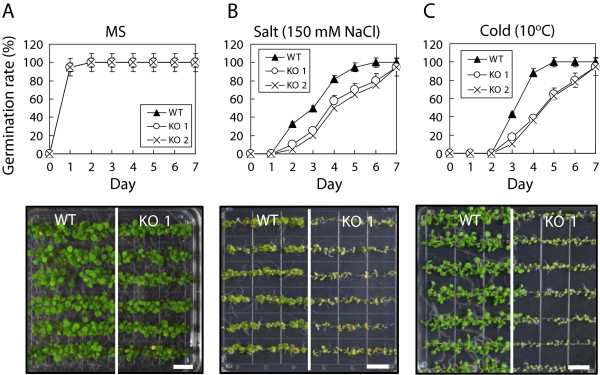
**Response of *cfm4 *mutant plants to abiotic stresses.** Seed germination and seedling growth of wild-type plant (WT) and *cfm4* mutants **(A)** on MS medium, **(B)** on MS medium supplemented with 150 mM NaCl, or **(C)** on MS medium at 10°C. Germination rate was scored on the indicated days. The pictures were taken 16 days after germination. Scale bar = 1 cm.

To determine whether CFM4 plays a role in the plant response to environmental stresses, the wild-type, *cfm4* mutants, and complementation lines were grown in MS medium supplemented with NaCl for salt stress or with mannitol for dehydration stress, or the plants were grown at 10°C for cold stress treatment. We first compared seed germination rates of the plants under normal and stress conditions. No differences were observed in seed germination rates between the wild-type and mutant plants under normal conditions (Figure 4A). However, seed germination of *cfm4* mutants was retarded compared with that of the wild-type and complementation lines under salt or cold stress conditions (Figure 4B and 4C), whereas seed germination rates of all three genotypes were similar to each other under dehydration stress conditions (Additional file 6). Seedling growth of *cfm4* mutants was also retarded compared with that of the wild-type and complementation lines under salt or cold stress (Figure 4). These results show that CFM4 affects seed germination and subsequent seedling growth of *Arabidopsis* under salt or cold stress conditions.

### CFM4 affects rRNA processing in chloroplasts

**Figure 5:**
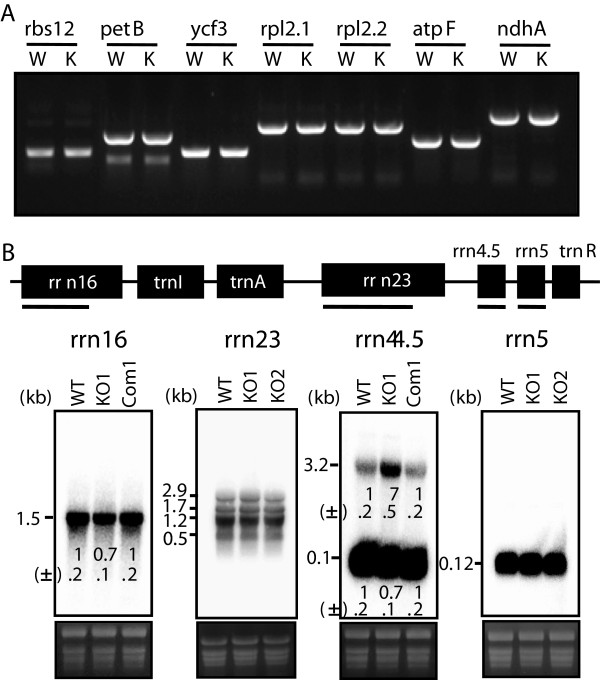
**Splicing patterns of chloroplast transcripts and rRNA processing in *cfm4 *mutant plants. (A)** Total RNAs were extracted from 4-week-old wild-type (W) and *cfm4* mutant (K), and the levels of intron-containing chloroplast transcripts were analyzed by RT-PCR. Identical results were obtained from independent experiments, and a representative result is shown. **(B)** Total RNA was extracted from 4-week-old wild-type (WT), *cfm4* mutants, and complementation line (Com1) and was separated on a 1.2% formaldehyde agarose gel. The levels of the processed products of 23S, 16S, 5S, and 4.5S rRNAs were determined by Northern blot analysis using the probes corresponding to each gene, represented by thick lines below each gene. The relative intensities of rRNA bands in *cfm4* mutants and complementation lines compared with those in wild type were calculated and values under each lane are means ± SD (n = 4).

The CRM domain-containing proteins have been demonstrated to be involved in the splicing of group II introns of chloroplast mRNAs and tRNAs [13,16,18,19]. Therefore, we first examined whether CFM4 is involved in the splicing of intron-containing genes in chloroplasts. Splicing patterns of all intron-containing chloroplast transcripts, including 15 mRNAs and 6 tRNAs, were analyzed by RT-PCR. The results showed that the splicing patterns of all intron-containing chloroplast transcripts were not altered in *cfm4* mutants compared with those in the wild-type plants (Figure 5A and Additional file 7), suggesting that CFM4 is not involved in intron splicing in chloroplasts. We next examined whether CFM4 is involved in rRNA processing. The levels of rRNAs, including 23S, 16S, 5S, and 4.5S rRNAs, in the wild type, *cfm4* mutant, and complementation line were determined by Northern blot analysis. To accurately determine relative levels of rRNA transcripts, Northern blot analysis was repeated four times, and the relative intensities of rRNA bands in *cfm4* mutants and complementation lines compared with those in wild type were calculated. The results showed that the transcript levels of the 23S and 5S rRNAs in *cfm4* mutants were comparable with those in the wild-type plants. However, the mature-16S rRNA transcript levels in *cfm4* mutant decreased compared with those in the wild-type plants (Figure 5B). In addition, the levels of mature-4.5S rRNA decreased, whereas the levels of precursor-4.5S rRNA increased in *cfm4* mutant. These abnormal levels of 16S and 4.5S rRNAs observed in *cfm4* mutants were restored to wild-type levels in the complementation line (Figure 5B). These results indicate that CFM4 affects the processing of 16S and 4.5S rRNAs.

### CFM4 possesses RNA chaperone activity

**Figure 6:**
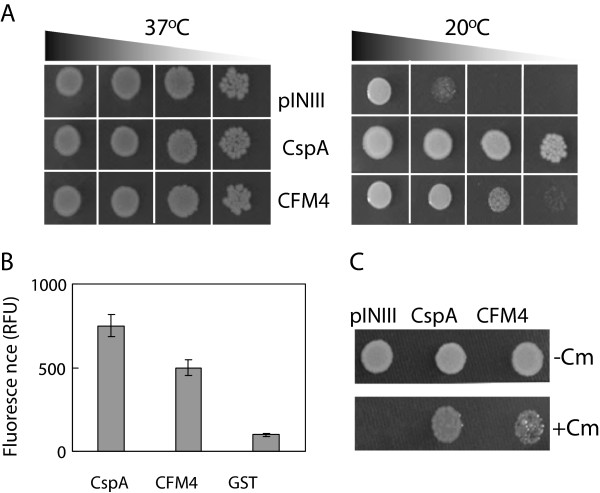
**RNA chaperone activity of the CFM4 protein. (A)** Complementation ability of CFM4 in *E. coli* BX04 mutant cells during cold shock. The diluted cultures of *E. coli* cells expressing CFM4, CspA (positive control), or pINIII vector (negative control) were spotted on LB-agar plates, and the cells were incubated at either 37°C or 20°C. **(B)** DNA-melting ability of CFM4. Fluorescence of the molecular beacons was measured after adding the recombinant GST-CFM4, GST-CspA (positive control), or GST (negative control) proteins. **(C)** Transcription anti-termination activity of CFM4. The *E. coli* RL211 cells expressing each construct were spotted on LB-agar medium with (+) or without (-) chloramphenicol (Cm), and the cells were incubated at 37°C.

RNA processing and intron splicing require proper folding of RNA substrates, and many RBPs that harbor RNA chaperone activity play an important role during these cellular processes [23-25]. Because CFM4 harbors a CRM domain that is known as an RNA binding module [13], we aimed to determine whether CFM4 possesses RNA chaperone activity. We first analyzed the complementation ability of CFM4 in the cold-sensitive *E. coli* BX04 mutant in which four cold shock proteins known as RNA chaperones are deficient and is high sensitivity to lower temperatures [26]. When the BX04 cells harboring each construct were incubated at 37°C, all cells grew well with no difference. However, when the BX04 cells were exposed to cold shock at 20°C, the cells expressing CFM4 or CspA as a positive control grew well at low temperatures, whereas the cells harboring the pINIII vector did not grow at low temperatures (Figure 6A). This result demonstrates that CFM4 has the ability to complement RNA chaperone-deficient *E. coli* mutant cells, suggesting that CFM4 functions as an RNA chaperone in *E. coli* under cold shock.

To further confirm whether CFM4 possesses RNA chaperone activity, the *in vitro* and *in vivo* nucleic acid-melting abilities of CFM4 were assessed via DNA-melting and transcription anti-termination assays. For the analysis of *in vitro* DNA-melting ability of CFM4, the recombinant GST-CFM4 fusion proteins were purified from *E. coli* (Additional file 8) and GST-CFM4 fusion protein was tested for its ability to destabilize base pairs in the synthetic DNA molecules labeled with a fluorophore (tetramethyl rhodamine) and quencher (dabcyl). Fluorescence of the molecular beacon increased after adding the GST-CFM4 proteins or the GST-CspA fusion proteins (positive control), confirming the DNA-melting activity of CFM4 (Figure 6B). By contrast, adding GST alone (negative control) did not increase fluorescence (Figure 6B). To determine whether CFM4 has the ability to destabilize base pairs in RNA, the *in vivo* RNA-melting ability of CFM4 was assessed via transcription anti-termination assays using *E. coli* RL211 cells that harbor a chloramphenicol resistance gene downstream from a *trpL* terminator with stem-loop structure [27]. When the RL211 cells were grown in the MS medium containing chloramphenicol, the cells expressing CFM4 or CspA grew well, suggesting that CFM4 and CspA destabilize the stem-loop structures present in the transcription termination signal. By contrast, the cells harboring the pINIII vector did not grow in chloramphenicol-containing MS medium (Figure 6C). Taken together, these results indicate that CFM4 possesses RNA chaperone activity.

## Discussion

The results presented here demonstrate that CFM4, one of two *Arabidopsis* genes encoding single CRM domain-containing protein, plays a role in the *Arabidopsis* growth and stress response by affecting rRNA processing in chloroplasts. Analysis of the T-DNA knockout lines indicated that CFM4 exhibits important function during plant growth and senescence (Figures 2 and 3), which is in agreement with its postulated roles in chloroplast RNA metabolism. Our current results point to the important roles of CRM domain-containing proteins in plant growth and development. It has been demonstrated that mutation in the genes encoding multiple CRM domain-containing proteins causes pale-green phenotypes, delayed development, and aborted seed production in plants [11,12,17,20,21]. In addition to its roles in the growth and senescence of *Arabidopsis* under normal growth conditions, CFM4 also affected the plant response to abiotic stresses. Seed germination and seedling growth of *cfm4* mutants were retarded compared with those of the wild-type plants under salt or cold stress conditions (Figure 4), suggesting that CFM4 plays a positive role in seed germination and seedling growth of *Arabidopsis* under salt or cold stress conditions. Interestingly, the effect of CFM4 on seed germination and seedling growth of plants was confined to salt or cold stress, as no differences in seed germination and seedling growth were observed between the wild type and *cfm4* mutants under dehydration stress conditions. Notably, the genes encoding single CRM domain-containing proteins as well as the genes encoding multiple CRM domain-containing proteins play important roles in plant growth, development, and stress response.

All multiple CRM domain-containing proteins whose functions have been characterized in plants are involved in the splicing of chloroplast group II and I introns [17]. CAF1, CAF2, and CRS1, the three maize proteins harboring multiple copies of the domain, are required for the splicing of group II introns in chloroplasts [15,28]. In addition, it has been demonstrated that CFM2 and CFM3 together with CRS2/CAF complexes promote the splicing of chloroplast introns [12,17]. In contrast to the well-characterized roles of multiple CRM domain-containing proteins in the splicing of chloroplast introns, CFM4 plays no role in the splicing of chloroplast introns (Figure 5A and Additional file 7). However, our current analysis shows that CFM4 is involved in the processing and maturation of 16S and 4.5S rRNAs (Figure 5B). This observation is in line with previous findings that prokaryotic proteins harboring a single CRM domain participate in ribosome maturation [13]. The YhbY, the CRM domain protein in *Escherichia coli*, is a small protein with molecular mass of ~10 kDa and is associated with pre-50S ribosomal subunits, which function in ribosome assembly [13]. Among the 16 CRM domain proteins found in *Arabidopsis*, CRM4 is the most homologous to YhbY in that it harbors little else except a single CRM domain (Additional file 1). We propose that the delayed growth of *cfm4* mutants is due to, at least in part, improper processing of 16S and 4.5S rRNAs in chloroplasts. Interestingly, improper rRNA processing in *cfm4* mutants resulted in impaired ABA biosynthesis (Figure 2), which supports a notion that proper chloroplast function is required not only for active photosynthesis but also for ABA biosynthesis, both of which are required for normal plant growth [22].

The proposed mechanistic role of CFM4 as an RNA chaperone in chloroplast rRNA metabolism is intriguing. RNA processing as well as intron splicing requires proper folding of RNA substrates, and many RBPs play an important role in RNA-RNA and RNA-protein interactions during RNA metabolism [23,29-31]. RNA chaperones are nonspecific RBPs that bind diverse RNA substrates and help RNAs fold by inducing structural rearrangement of misfolded RNAs [25,32,33]. It has recently been demonstrated that one of the minor spliceosomal proteins U11/U12-31 K possesses RNA chaperone activity and is indispensible for correct intron splicing and normal growth and development in *Arabidopsis* and rice [23,34], which emphasizes the important role of RNA chaperones in maintaining RNA substrates in splicing-competent structures for correct processing to occur. Involvement of RNA chaperone activity in the splicing of group I and group II introns has been demonstrated in yeast mitochondria. The yeast DEAD-box protein MSS116 promotes the splicing of both group I and group II introns in mitochondria via functioning as an RNA chaperone [35-38]. Our current analysis clearly indicates that CFM4 harbors RNA chaperone activity (Figure 6). It is likely that RNA chaperone activity of CFM4 is needed to maintain precursor-rRNA in processing-competent structures for subsequence rRNA processing. Although it is not clear at present how CFM4 affects processing of only two (16S and 4.5S) rRNAs out of the four rRNAs in the chloroplast, it is possible that CFM4 recognizes specific sequence or structural elements in 16S and 4.5S rRNAs and the RNA chaperone activity of CFM4 is involved in the formation of processing-competent structures of these rRNAs.

## Conclusions

The present study shows that CFM4 harboring RNA chaperone activity is involved in rRNA processing in chloroplasts, which is important for growth and the stress response of plants. Although much progress has been made in the characterization of the roles of CRM domain-containing proteins in intron splicing and rRNA processing during chloroplast gene expression, the roles of many CRM proteins in plant growth and development as well as chloroplast RNA metabolism remain largely unknown. In particular, understanding the biological function of single CRM domain-containing proteins is far behind compared with that of multiple CRM domain-containing proteins. Exploring the importance of the number of CRM domains and involvement of each CRM domain in substrate recognition during intron splicing and rRNA processing should provide a much deeper insight into the mechanistic role of CRM family members in chloroplast RNA metabolism.

## Methods

### Plant materials and growth conditions

The *Arabidopsis thaliana* used in this study was Col-0 ecotype. The plants were grown either in a mixture of vermiculite, peat moss, and perlite or on half-strength Murasige & Skoog (MS) medium containing 1% sucrose at 23 ± 2°C under long-day conditions (16 h light/8 h dark cycle). To construct overexpressing transgenic plants and complementation lines, the CFM4 cDNA coding sequence was cloned into the *Nco*I/*BstE*II of pCambia1301 vector containing the cauliflower mosaic virus 35S promoter. The *Arabidopsis* transformation was carried out via vacuum infiltration [39] using *Agrobacteruim tumefaciens* GV3101. The T_3_ transgenic lines were selected and subsequently utilized for the phenotype investigation. Expression of *CFM4* in each transgenic plant was analyzed by RT-PCR using the gene-specific primers listed in Additional file 9. *Arabidopsis* mutant seeds with T-DNA inserted into the CFM4 gene (SALK_076439 and SALK_126978) were obtained from The Arabidopsis Biological Resource Center (Columbus, OH, USA). The absence of *CFM4* expression in the mutant lines was confirmed by RT-PCR using the gene-specific primers listed in Additional file 9.

### Analysis of cellular localization of CFM4 in *Arabidopsis*

To determine the cellular localization of CFM4, the cDNA corresponding to the full-length CFM4 was ligated using the *Xba*I/*BamH*I site in front of the GFP gene, and CFM4-GFP fusion proteins were expressed under the control of the CaMV 35S promoter in *Arabidopsis*. The GFP signals in the leaves and roots of the plants were observed using a Zeiss LSM510 laser scanning confocal microscope (Carl Zeiss Inc. Thornwoold, NY, USA). The excitation and emission wavelengths were 488 and 545 nm, respectively. Mitochondria in the leaves and roots of the plants were stained with MitoTracker® Red CMXRos (Invitrogen).

### RNA extraction and RT-PCR

Total RNAs were extracted from frozen tissues using the Plant RNeasy Extraction kit (Qiagen, Valencia, CA, USA), and the concentration of RNAs was quantified by spectrophotomety. RT-PCR was performed using the primers spanning the T-DNA insertion site to confirm whether the T-DNA tagged mutant seeds from ABRC were knockout mutants. To examine splicing patterns of intron-containing genes, total RNAs were extracted from 4-week-old plants, and 100 ng RNA was used for RT-PCR with the gene-specific primers listed in Additional file 9. To examine the expression levels of ABA biosynthesis-related genes, total RNAs were extracted from 26-day-old plants, and 100 ng RNA was used for quantitative real-time RT-PCR with the gene-specific primers listed in Additional file 9.

### Measurement of ABA content

Ten grams of plant tissues at 26 DAG were ground in liquid nitrogen, mixed with an extraction solution (80% methanol, 2% acetic acid), and incubated overnight at 4°C. After centrifugation at 2000 × g for 10 min, the pellet was dissolved in 10% methanol and TBS (50 mM Tris, 0.1 mM MgCl_2_, and 0.15 M NaCl, pH 7.8). ABA concentration was determined using Phytodetek® ABA Test kit (Agdia Inc., Elkhart, Indiana, USA) according to the manufacturer’s instructions.

### Northern blot analysis of rRNA transcripts in chloroplasts

Total RNA was extracted from 4-week-old wild-type and *cfm4* mutant plants for chloroplast rRNA processing analysis*.* Five or ten micrograms of total RNA was electrophoresed on an 1.2% agarose or 16.5% formaldehyde (w/v) gel, blotted to a Hybond-N^+^ nylon membrane (Amersham Biosciences, Parsippany, NJ, USA), and then cross-linked under UV. Hybridization with ^32^P-labeled probes was performed in 0.15% SDS, 5 × SSC, 5 × Denhardt’s solution, salmon sperm DNA (10 mg/ml) and 50% formamide at 42°C overnight. The membrane was washed 2-3 times in 2 × SSC and 0.1% SDS at room temperature and once in 0.1 × SSC and 0.1% SDS at 42°C. The genes encoding chloroplast rRNAs were cloned into the pGEM-T Easy vector using the *Arabidopsis* chloroplast sequence information (GenBank accession no. AP00023), and the hybridization probes were amplified by PCR from the chloroplast rDNA-cloned vector. The probes contained rDNA sequences of the following region: rrn16S (position 101,500-102,482), rrn23S-5′ (position 104,691-106,691), rrn23S-3′ (position 106,692-107,500), rrn4.5S (position 107,599-107,701), and rrn5S (130,580-130,700). The probes were labeled with ^32^P-dCTP using the Random Primer DNA Labeling Kit (TaKaRa Bio., Shiga, Japan) according to the manufacturer’s instructions.

### RNA chaperone assay

For the cold shock and transcription anti-termination assays in *E. coli,* the pINIII vector expressing CFM4 was constructed essentially as described previously [40]. In the cold shock assay, the vector was introduced into *E. coli* BX04 mutant cells [26] that lack four cold shock proteins and thus are highly sensitive to cold stress*.* The BX04 mutant cells harboring either pINIII-CFM4, pINIII-CspA (positive control), or pINIII (negative control) were grown in Luria-Bertani (LB) medium containing ampicillin and kanamycin, and the serial-diluted cultures (from 10^-1^ to 10^-5^) of BX04 cells were spotted on LB medium and incubated at low temperature (20°C). In the transcription anti-termination assay, *E. coli* RL211 cells [27] transformed with each construct were grown in liquid LB medium and spotted on LB-carbenicillin plates with or without chloramphenicol. Growth of the cells was inspected on a daily basis.

For *in vitro* DNA-melting assay, the DNA molecules labeled with a fluorophore (tetramethyl rhodamine) and quencher (dabcyl) were synthesized as previously described [40,41] For the expression and purification of recombinant GST-CFM4 fusion proteins in *E. coli*, the coding region of CFM4 was cloned into pGEX-5X-2 vector (Amersham Pharmacia Biosciences). The recombinant GST-CFM4 fusion proteins were expressed in BL21 DE3 competent cells (Promega) and purified using glutathione Sepharose 4B resin. The fluorescence emitted from the reaction between the GST-CFM4 fusion proteins and the DNA molecules was measured using a Spectra Max GeminiXS spectrofluorometer (Molecular Devices, Sunnyvale, CA, USA) with excitation and emission wavelengths of 555 nm and 575 nm, respectively.

## Abbreviations

CRM: Chloroplast RNA splicing and ribosome maturation; CFM4: CRM family member subfamily 4; DAG: Day after germination; RBP: RNA-binding protein.

## Competing interests

The authors declare that they have no competing interests.

## Authors’ contribution

KL and HK designed the experiments; KL, HJL, DHK and YJ conducted most of research and analyzed the data together with HSP and HK; KL and HK contributed to the writing of the manuscript. All authors read and approved the final manuscript.

## Supplementary Material

Additional file 1 Alignment of the amino acid sequences of a single CRM domain-containing proteins from various plant species. Click here for file

Additional file 2 Confirmation of knockout mutants and complementation lines. Click here for file

Additional file 3 **Phenotypes of *cfm4 *** **mutant plants.** Click here for file

Additional file 4 **Phenotypes of *cfm4 *** **mutant plant and complementation line.** Click here for file

Additional file 5 **Root growth of *cfm4 *** **mutant and complementation lines.** Click here for file

Additional file 6 **Response of *cfm4 *** **mutant plants to dehydration stress.** Click here for file

Additional file 7 **Splicing patterns of chloroplast transcripts in *cfm4 *** **mutant plant.** Click here for file

Additional file 8 **Purification of recombinant glutathione S-transferase CFM4 fusion protein in *E. coli.***
Click here for file

Additional file 9: Table S1 Gene-specific primer pairs used in RT-PCR experiments. Click here for file
