# Supplementary material for: A nuclear-encoded chloroplast protein harboring a single CRM domain plays an important role in the Arabidopsis growth and stress response
Source: BMC Plant Biol. 2014 Apr 16;14:98. doi: 10.1186/1471-2229-14-98 (PMC4021458; doi:10.1186/1471-2229-14-98)
Supplement: Additional file 1 — Alignment of the amino acid sequences of a single CRM domain-containing proteins from various plant species. [file 1471-2229-14-98-S1.doc]

**Additional file 1.** Alignment of the amino acid sequences of a single CRM domain-containing proteins from various plant species. The alignment was made using the ClustalW program. Gaps in the sequences are indicated by dashes. CRM domain is indicated by thick line, and the position of the conserved GxxG sequence is indicated by red box. Accession numbers for protein are as follows; *A. thaliana*1 (CFM4), At4g39040; *A. thaliana*2, At2g21350; *G. max*, XP_003537849; *V. vinifera*, XP_002265886; *M. truncatula*, XP_003607024; *H. vulgare*, BAJ90757; S. bicolor, XP_002464382; *O. sativa*, Os10g36860; *E. coli* YhbY, JW3147.
