# Supplementary material for: A nuclear-encoded chloroplast protein harboring a single CRM domain plays an important role in the Arabidopsis growth and stress response
Source: BMC Plant Biol. 2014 Apr 16;14:98. doi: 10.1186/1471-2229-14-98 (PMC4021458; doi:10.1186/1471-2229-14-98)
Supplement: Additional file 2 — Confirmation of knockout mutants and complementation lines. [file 1471-2229-14-98-S2.doc]

**Additional file 2.** Confirmation of knockout mutants and complementation lines. (A) Schematic presentation of T-DNA insertion site of KO1 (SALK_076439) and KO2 (SALK_126978) mutant lines. Exons are indicated by black rectangles and introns are indicated by black lines. The 5’- and 3’- untranslated regions are indicated by white rectangles. The site of T-DNA insertion in CFM4 was shown by a filled black triangle, and the arrow indicates the direction of left border. (B) The transcript levels of *CFM4* in Col-0, mutants (KO1 and KO2), and complementation lines (Com1 and Com2) were determined by RT-PCR
