# Supplementary material for: A nuclear-encoded chloroplast protein harboring a single CRM domain plays an important role in the Arabidopsis growth and stress response
Source: BMC Plant Biol. 2014 Apr 16;14:98. doi: 10.1186/1471-2229-14-98 (PMC4021458; doi:10.1186/1471-2229-14-98)
Supplement: Additional file 3 — Phenotypes of cfm4 mutant plants. [file 1471-2229-14-98-S3.doc]

**Additional file 3.** Phenotypes of *cfm4* mutant plants. The growth of the wild-type (WT) and *cfm4* mutants (KO1 and KO2) at (A) 7 days and (B) 23 days after germination (DAG). Scale bar = 1 cm.
