# Supplementary material for: A nuclear-encoded chloroplast protein harboring a single CRM domain plays an important role in the Arabidopsis growth and stress response
Source: BMC Plant Biol. 2014 Apr 16;14:98. doi: 10.1186/1471-2229-14-98 (PMC4021458; doi:10.1186/1471-2229-14-98)
Supplement: Additional file 4 — Phenotypes of cfm4 mutant plant and complementation line. [file 1471-2229-14-98-S4.doc]

**Additional file 4.** Phenotypes of *cfm4* mutant plant and complementation line. The growth of the wild-type (WT), *cfm4* mutant (KO2), and complementation line (Com2) at 33 days after germination. Scale bar = 1 cm.
