# Supplementary material for: A nuclear-encoded chloroplast protein harboring a single CRM domain plays an important role in the Arabidopsis growth and stress response
Source: BMC Plant Biol. 2014 Apr 16;14:98. doi: 10.1186/1471-2229-14-98 (PMC4021458; doi:10.1186/1471-2229-14-98)
Supplement: Additional file 5 — Root growth of cfm4 mutant and complementation lines. [file 1471-2229-14-98-S5.doc]

**Additional file 5.** Root growth of *cfm4* mutant and complementation lines. The wild-type (WT), *cfm4* mutant (KO1), and complementation lines (Com1 and Com2) were grown in MS medium at vertical orientation. Root lengths of the plants were measured at 12 days after germination. Asterisk above the column indicates values that are statistically different from the control (WT) values (p ≤0.05). Scale bar = 1 cm.
