# Supplementary figures and images for: A nuclear-encoded chloroplast protein harboring a single CRM domain plays an important role in the Arabidopsis growth and stress response
Source: BMC Plant Biol. 2014 Apr 16;14:98. doi: 10.1186/1471-2229-14-98 (PMC4021458; doi:10.1186/1471-2229-14-98)

## Slide 1
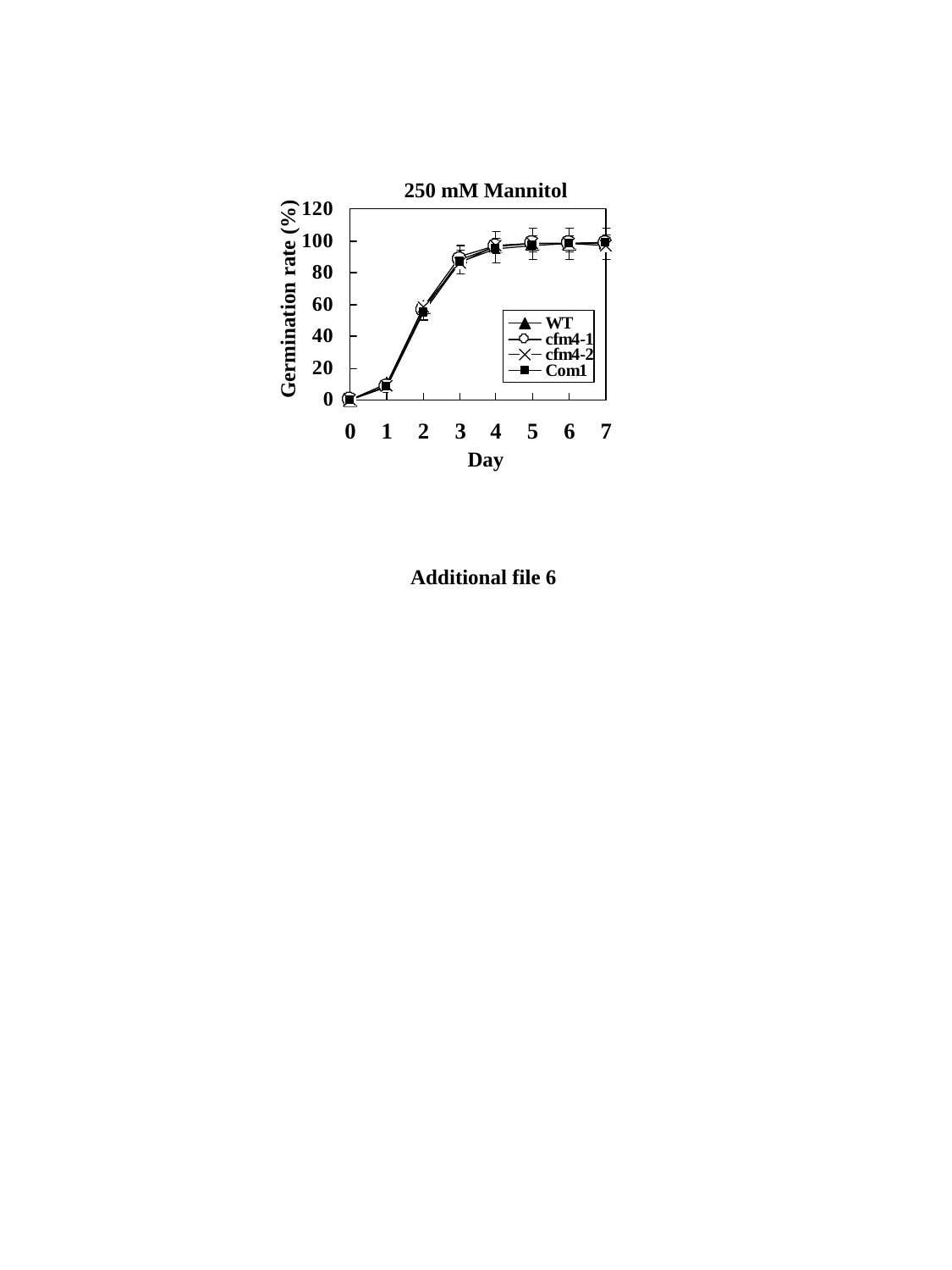

250 mM Mannitol
Germination rate (%)
Day
 Additional file 6

Supplement: Additional file 6 — Response of cfm4 mutant plants to dehydration stress. [file 1471-2229-14-98-S6.pptx]
