# Supplementary material for: A nuclear-encoded chloroplast protein harboring a single CRM domain plays an important role in the Arabidopsis growth and stress response
Source: BMC Plant Biol. 2014 Apr 16;14:98. doi: 10.1186/1471-2229-14-98 (PMC4021458; doi:10.1186/1471-2229-14-98)
Supplement: Additional file 7 — Splicing patterns of chloroplast transcripts in cfm4 mutant plant. [file 1471-2229-14-98-S7.doc]

**Additional file 7.** Splicing patterns of chloroplast transcripts in *cfm4* mutant plant. Total RNAs were extracted from 4-week-old wild-type (W) and *cfm4* mutant (K), and the levels of intron-containing chloroplast transcripts were analyzed by RT-PCR. Identical results were obtained from independent experiments, and the representative results are shown.
