# Supplementary material for: A nuclear-encoded chloroplast protein harboring a single CRM domain plays an important role in the Arabidopsis growth and stress response
Source: BMC Plant Biol. 2014 Apr 16;14:98. doi: 10.1186/1471-2229-14-98 (PMC4021458; doi:10.1186/1471-2229-14-98)
Supplement: Additional file 8 — Purification of recombinant glutathione S-transferase CFM4 fusion protein in E. coli. [file 1471-2229-14-98-S8.doc]

**Additional file 8.** Purification of recombinant glutathione S-transferase CFM4 fusion protein in *E.coli.* CspA, GST and CFM4 were expressed in BL21(DE) by adding 0.2 mM of IPTG, anaylyzed through SDS-PAGE, and purified by gluthation resin. M: molecular mass marker.
