# Supplementary material for: A nuclear-encoded chloroplast protein harboring a single CRM domain plays an important role in the Arabidopsis growth and stress response
Source: BMC Plant Biol. 2014 Apr 16;14:98. doi: 10.1186/1471-2229-14-98 (PMC4021458; doi:10.1186/1471-2229-14-98)
Supplement: Additional file 9: Table S1 — Gene-specific primer pairs used in RT-PCR experiments. [file 1471-2229-14-98-S9.doc]

**Additional file 9: Table S1.** Gene-specific primer pairs used in RT-PCR experiments
